# The supernatural characters and powers of sacred trees in the Holy Land

**DOI:** 10.1186/1746-4269-3-10

**Published:** 2007-02-25

**Authors:** Amots Dafni

**Affiliations:** 1 Institute of Evolution, the University of Haifa, Haifa 31905, Israel

## Abstract

This article surveys the beliefs concerning the supernatural characteristics and powers of sacred trees in Israel; it is based on a field study as well as a survey of the literature and includes 118 interviews with Muslims and Druze.

Both the Muslims and Druze in this study attribute supernatural dimensions to sacred trees which are directly related to ancient, deep-rooted pagan traditions. The Muslims attribute similar divine powers to sacred trees as they do to the graves of their saints; the graves and the trees are both considered to be the abode of the soul of a saint which is the source of their miraculous powers. Any violation of a sacred tree would be strictly punished while leaving the opportunity for atonement and forgiveness. The Druze, who believe in the transmigration of souls, have similar traditions concerning sacred trees but with a different religious background.

In polytheistic religions the sacred grove/forest is a centre of the community's official worship; any violation of the trees is regarded as a threat to the well being of the community. Punishments may thus be collective.

In the monotheistic world (including Christianity, Islam and Druze) the pagan worship of trees was converted into the worship/adoration of saints/prophets; it is not a part of the official religion but rather a personal act and the punishments are exerted only on the violating individual.

## Background

In many religions, sacred places [[1]:90,235,254; [2]:85; [3]: 399,171; [4]: passim; [5]:passim], objects [[6]:106;[7]:534; [8]:169,176,179], as well as saints [[9]:184; [10]:2; [11]:121,129,131; [12]:72,85] were thought to possess supernatural characters and the power to carry out miracles or as having magical powers. The same attitude is found throughout history as an outcome of tree worship/adoration/veneration [[13]: passim; [14]: 42–45; [15]: 210–211,215; [16]:40; [17]: 23; [18]: passim; [19]: passim; [20 passim; [21]:68–70; [22]:72–79; [23]: passim].

Sacred trees were sometimes described as possessing huge or unusual dimensions or miraculous physical characters [[20]: passim; [17]:8, 23; [15]:215, [24]:339; [25]:35; [26]:38]. Frequently, sacred trees were regarded as having omnipotent magic powers to punish, cure, or to ccarry out miracles and to confer unusual abilities [[14] 14, 42–45; [23]: passim; [13]: passim; [19]:14–17; [18]:23; [20]:32,35,41] see also Tables 1 and 2.

**Table 1:** The supernatural characters of sacred trees

Character	Druze (n = 27)	Arabs (n = 24)	Bedouin (n = 34)	References from the Middle East	References from other regions
1. The tree is the abode of the soul of a righteous person	0	83.3	88.2	[Palestine (40:15); (42: passim)].	
2. Unusual lights/voices appear around the tree	22.2	20.0	11.7		
3. The tree is not burned	22.2	12.5	17.6	[Ancient Semites (61:1930); Palestine (87:66), (167:1860),(400:73); Arabia (15:215)]	[Ireland (20:38); Morocco (26:142); Japan (25:35)]
4. Miracles related to the tree	22.2	20.8	11.7		[Estonia (18:23); Ireland (20:32,35,41); India (90:220),(168:294)]
5. The tree bleeds when injured/cut	22.2	0	5.8	[Israel, Bedouin (68:89–90); Iraq, Mandeans, (169:348)];	[Rome (39: 738–878); Morocco (52:78); Cameroon (170:82); India (171:40), (156:66)].
6. The tree has unusual dimensions	0	11.1	0	Persia (Williams-Jackson, 1965 172:263)	Japan (24:339)]
7. The tree has unusual/miraculous fruit/leaves	18.4	11.1	0		[Ireland (20:18–20); Central Asia (89:350)]
8. sacred tree is not eaten by locust	0	11.1	0	[Palestine (40:36)]	
9. Trees as oracles	0	0	0	[Ancient Semites (61:195), (73:78); Arabia (74, II:209)]	[Ancient Greece (72:passim), (17: passim), (50:58); Rome (50:59), (27:274)]

**Table 2:** The supernatural powers of sacred trees.

The supernatural power	Druze (n= 27)	Arabs (n= 24)	Bedouin (n = 34)	References from the Middle East	References from other regions
1. Punishing of the tree violators	88.8	91.6	91.1	See Table 3	See Table 3
2. Granting a divine blessing (*barake*); petitions for health/wishing tree/tree as mediator to God	77.7	91.6	82.3	[Palestine (40:107:108), (41: passim) (42: passim), (111:48,51)]	[Estonia (173:4); Sierra Leone (174:48); East Africa (175:4); Chad (176; 248); Japan (177:23), (16:10); Australia, New Zealand (23:164)]
3. Breakdown of machinery or vehicles	47.0	62.5	41.1	[Palestine (111:69)]	[India (90: 219–220)]
4. Punishing for false oaths	14.8	44.4	23.5		[India (186:266)]
5. Protection of properties deposited underneath.	22.2	25.0	29.4	[Palestine (43:215), (40:102), (178:138)]	
6. Cure of ill domestic Animals (circumambulation around the tree)	33.3	8.3	2.9		[Scotland (179, II:184–185)]
7. Parts of the tree used as medicine	29.6	20.8	17.6	Palestine (87: 66);, Egypt (46:56)]	[Pagan Europe (35:276) East Africa (180:278, 279)]
8. Parts of the tree used as a talisman	22.2	12.5	11.7	[Palestine (181:129); Egypt (182:11)]	Ancient Celts (51:199); Ireland (20:36,38); East Africa (Kikuyu, 175:5); India (27:590,(6:24)]
9. Food/objects are placed under the tree to absorb the *barake*	18.5	25.0	11.6	[Palestine (111:52)]	
10. Shelter from violence/revenge/	18.5	16.6	11.7		[Ancient Greece (53:173, (37:335), (53:173), (13:51), (17:221–228); Rome (133: passim),(50:68); Ghana (183:149), (184;129) India (100:241,252)]
11. Sleeping/resting under the tree as a *barake*/medicine	14.8	0	0	[Palestine (74:I:448); Arabia (14:50), (91:331), (185:30)]	[Many countries (179, II:169–173)]
12. Crawling under the tree/through the split in the trunk for a cure	0	0	0	Lebanon (122:16),(67:2430; Syria (28: 179)]	Ancient Celts (51:331); Pagan Europe (179, II: 173,169–171), (14:50); Britain (187:129)]

In many cultures around the globe sacred trees are strictly protected, and injuring the tree in any way is regarded as sacrilege. This deep faith has been established in the course of generations by tradition and stories of actual punishment meted out by the gods/souls/saints/demons to which the trees are dedicated [[13]:24–52; [27]:68–69; [28]:316; [227]:243; [30]:146, 77; [25]:37; [31]:97]. Injuring the tree, especially cutting it down may leave the resident spirit homeless and it would soon take its revenge. This is the reason why people are afraid to harm sacred trees [[32]:99; [33]: I: 133; [34]:148].

In Greece and Rome sacred groves were protected by gods and there were strict regulations against any violation of these groves [[35]:227; [17]: passim; [36]:442–443; [37]:334–335; [38]: 59–66, 73–77]. According to Ovid, Erysichton cut down a sacred tree devoted to Demeter and was punished by an everlasting and insatiable hunger [[39]: 741].

In the Middle East, in particular, sacred trees are regarded as the abode of the souls of local saints known as Wellis [[28]: 316; [40]:151; [41]: 322; [42]: passim]. In this region and in North Africa sacred trees are protected by the Wellis to whom they are dedicated [[40]:36; [43]:205; [44]:68–69; [45]:385; [42]: passim]. The Welli confers his divine powers on the tree, which acts on humans [[40]: 35–38; [28]: 316; [41]: passim; [42]: passim]; any hurt/damage to this tree is regarded as a personal insult against the saint, who will promptly/immediately retaliate to defend his reputation and/or territory [[40]:102; [14]:42].

In some communities fear of revenge by the spirits in response to any harm to the sacred tree is so great that special ceremonies, which may include sacrifices, ex-voto gifts, and/or prayers/ceremonies, are held to pacify the angry spirit before a tree is cut down [[14] I:133–137; [46] 15:56; [25]:36; [47]:229; [48]:23; [49]:313]. This custom is already known from ancient Greece [[17]: passim; [38]:59–66] and Rome [[32]:99; [50]:76; [38]: 73–77].

Not surprisingly, any kind of harm done to the sacred tree/grove/forest is feared to cause a furious punitive response by a supernatural being [[13]: passim; [14]:42–45; see Table 4). This fear is so deeply imprinted in human conscience of various tribes/communities that the people are afraid even to pick a leaf [[51]:103,203; [50]:81; [20]:24;[52]:75,98) or to collect dead wood from below the tree [[53]:172; [54]:174; [50]:26; [20]:38,40; [55]:277, see also Table 3), which may carry an immediate sentence of death (Table 4).

**Table 3:** Punishable deeds in connection with sacred trees.

	Druze (n = 32)	Arabs (n = 31)	Bedouin (n = 28)	References from the Middle East	References from other regions
Tree injury/cutting down	68.7	58.8	89.2	[Palestine (14^th ^century (40:36), (45:376),(63:92), (128:179); Arabia (:28:316); Turkey (116:237)]	[Ancient Greece and Rome (,53:172), (36:44) Ancient Celts (51:162); Ancients Balts (188:159); Old Prussia and Lithuania (60, I:77); Estonia (50:166); Nigeria (176:258); Kenya (189:28); Tanzania (190:60); India (88:90), (90:218), (100:242), (137:345), (191:8); Nepal (192:248); Mongolia (77:280); Siam (193:195); Sri-Lanka (194:203,217); China (195: 332)]
1. Theft of property deposited under the tree.	3.1	61.2	78.5	Palestine (40:102)]	
2. Collecting of dead wood	32.3	32.2	28.5	[Palestine (40:3600, (111:43) (126:28), (128:179), (178:76); Egypt (132:17); Turkey (116:238)]	Ancient Greece (53:172), (134:157); Rome (50:26); Ireland (20:38,40); Iran (196:141); India (54:174); (55:277), (197:453); Ghana (50:22); Tanzania (198:45); Kenya (89:28); China (199:30;66)]
3. Picking of even one leaf	31.5	48.3	21.4	[Palestine (13^th ^century, (200:64), (45:304); Arabia (91:330)]	[Ancient Celts (51:103,203); Estonia (50:81); Ireland (20:24); Morocco (52:75,198); Tanzania (201:3); Madagascar (202:976); India (139:67); Sri Lanka (194:115);Timor (161:93)]
4. Road construction near the tree	25.0	38.7	25.07		[Japan (25:36)]
5. Ban on grazing near a sacred tree	24.2	24.5	35.7		[Ancient Greece (50:640, (37:334); Ireland (20:34); Mediterranean and India (54:174); Kenya (189:28); Tanzania (190:6)]
6. Disrespectful behaviour	15.6	19.3	10.7		Sri Lanka (194:115)]
7. Fruit harvesting	12.5	9.6	17.85	[Palestine, (40:35–36)]	[India; (88:95), (203:5); Samoa (186:265)]
8. Disturbing prayers	11.2	0	0		

**Table 4:** Punishments on the violation of sacred trees.

**Punishment**	Druze (n = 20)	Arab (n = 31)	Bedouin (n = 44)	References from the Middle East	References from other regions
1. Divine punishment, serious illness, disaster, accidents, bad luck, madness, curse	50.0	25.7	15.8	[Egypt (132:17); Turkey (116:175)]	[Greece (37:335); Britain (65:21); Ireland (20:21,33,47); Estonia (18:23), (212:3); Zanzibar (213:36); Mongolia (77:281); Siberia (216:117); Zimbabwe (83:6); Kenya (189:28); East Africa (48:432–433); Tanzania (198:27, 45); Zimbabwe (215:365); Nepal (192:248); (217:335); India (27:68–69), (30:73), (153:8),(154:201), (139:67); Sri Lanka 165:177); China (50:168), (214:746), (195:332); Japan (25:36)]
2. Paralysis (especially of the hand/limb fracture)	35.0	35.4	27.2	[Syria (112:16); Arabia (74:496)]	[Britain (65:22,191); Ireland (20:37,47); Lithuania (218:31)]
3. Damage to property, fire, death of a domestic animal	35.0	12.9	40.1	[Syria (112:16); Egypt (132:17); Palestine (111:44), (126:20); Sinai (110:185); Turkey (15;215]	[Ghana (160:259); Nepal (217:335); Estonia (18;23); India (100:240)]
4. Death, murder	12.0	35.4	9.0	Palestine (87:66); Syria (112:16); [Transjordan (74, I:77, 365); Arabia (91:330): Iran (219:118), (220:3); Kurdistan (15:216)]	[Ancient Greece (204:38,60, (50:61); Ireland (20:29,47); Britain (65:191); Lithuania (50:25); Estonia (18:23); Dalmatia (22:64); Siberia (216:117); Mongolia (77:281)] Cameroon (170:82); Ghana (161:159); Zanzibar (213:36); Nigeria (221:272); India (31:97), (55:277), (100:240), (153:8), (222:316); Indonesia (223:3, 4); Japan (25:36), (136:46)]
5. Death/illness of a family member	20.0	12.9	20.4	[Palestine (87:66); Arabia (74:496); Egypt (46:56); Iran (224:124); Turkey (15:215)]	[India (197:295); Nepal (217:335)]
6. Barrenness	15.0	6.4	2.2		[India (88:54)]
7. Blindness	15.0	0	0		
8. Fine, ransom, sacrifice, special gift	0	0	0	[Palestine [40]:35,36]	Ancient Greece (37:335); Rome (50: 67); Siberia (225:47); Ghana (160:159); Kenya (189:28); Nigeria (221:205); Africa (Bantu, 205:41); India (90:221), (138:107), (226:570)]
9. Collective punishment for the whole community	0	0	0		[India (156:21,83,96); China (152:131), (211:145); Vietnam (147:113); Laos (148:84)]

In previous papers we studied the reasons and the religious background why trees became sacred with a special reference to the Middle East [42]. In other papers we studied the custom of tying rags on sacred trees in Israel [41] and the special respect given to *Ziziphus spina-christi *[69]in our region. The present paper is a continuation of our studies in which we surveys the present-day attitudes of Muslims and Druze in Israel in relation to the supernatural characters and powers of the sacred tree.

## Methodology

The field study (1999–2005) centred on thirty-one Arab, Bedouin, and Druze villages in the Galilee. Informants were asked about the supernatural characters and powers of the sacred trees. The survey covered 118 informants, consisting of 38 Druze and 80 Muslims (36 Arabs and 44 Bedouins). We took "Arabs" to be people settled in their villages for several centuries; "Bedouin" people who originated in the deserts of Israel and Jordan, migrated to the Galilee in the last three centuries, and were nomads until the end of the 20^th ^century [[56]:30]. The Druze are an eastern Mediterranean religious group established in Egypt in the 11^th ^century [[57]:3]. Today they are concentrated in Lebanon, Syria, and Israel [[57]:8–14]; belief in the revelation of God in the form of a human being is considered the most important fundamental principle of the Druze faith [[57]:15]. The Druze faith is not a ritual and ceremonial belief in essence, but rather a neo-platonic philosophy [[57]:17].

The distinction between "Arabs" and "Bedouin" was made in an attempt to examine if there were any different traditions regarding sacred trees which may reflect the different origin of nomads versus settled village people.

The survey excluded Christians, who hardly believed in sacred trees while, in the Jewish sector, the adoration/worshipping of trees is a new trend of the last two decades and almost all the worshippd trees are already known as old Muslim sacred ones in the vicinity of graves of supposed historical righteous Jewish personalities. In each village we carried out a preliminary survey to locate the more knowledgeable people in advance, and we also chose important religious leaders to examine their attitudes to the veneration of sacred trees.

The informants were mainly chosen according to their knowledge of common/local traditions and/or religious status. The average age of the informants was 57.7 (+/- 14.8) years. Respondents were 116 males and two females (in general women are reluctant to be interviewed, and when they agreed the interview was held in the presence of other family members). Because of the refusal of most of the informants to be videotaped or recorded the study is based entirely on oral interviews and field notes taken on the spot. The interviewees were asked several specific questions; for example, 1. What is the reason for the specific punishment inflicted on anyone who has hurt/damaged/cut down/used the sacred tree? 2. Who is liable to be punished for injuring a sacred tree, and how may the punishment be averted or revoked? 3. Why are sacred trees not burned? 4. Who is the punishing agent in response to not respecting the sacred tree? We also collected stories of miracles related to sacred trees.

## Results

The results concerning the supernatural characters of sacred trees appear in Table 1; supernatural powers of sacred trees are in Table 2; punishable deeds in connection with sacred trees are shown in Table 3 and the punishments for those who violated sacred trees are presented in Table 4).

The questions that were asked, and the various answers given, are listed here.

A. What is the reason for the specific punishment inflicted on anyone who harmed/cut down/used the sacred tree? (Figures in bold type indicate the number of the informants who gave a particular answer).

1. The house fell down because the person took wood for constructing a house. (**5)**.

2. The house/food/property (of the wrong-doer) was burned because wood was taken for heating or burning. (**7)**

3. A leg was cut off, just as the tree was cut down. (**4)**

4. The same axe that cut down the tree cut the leg off/killed the offender. (**5**, all of whom are Druze).

B. Who is liable for punishment for harming a sacred tree, and how may the punishment be averted or revoked?

1. The person has to sacrifice a goat and to give its meat to the needy (**12**).

2. The material (such as leaves, branches) that was taken from the tree has to be returned (7).

3. Pilgrimage to a sacred place, sacrifice and/or the paying of a ransom (**5**).

4. The person to be punished can go to the Sheikh who reads a chapter from the Koran and the offender has to ask for forgiveness; the punishment will disappear instantly (**6**).

5. The offender expresses remorse and swears not to repeat his deeds (**4**).

6. Only believers are punished by the tree (**5)**.

7. If you coming to the sacred tree with a good intent, the Welli will bless you; if you are coming with an evil intent, you will be punished (**6**).

8. Even animals that ate from or touched the sacred trees or the seeds that fell nearby are mortally punished (**6**).

C. Why are sacred trees not burned?

1. The tree is protected by the Welli (**23**, all the ethnic groups))

2. The tree is protected by God (**11**, only one Druze)

3. "God gives power to the sacred tree so it does not burn". (Subhi Daoud, Muslim, Majdal Kurum, 10.10.01).

4."The tree enjoys divine protection because the prophet sat beneath it; after the death of the prophet Nobody can cut it down or burn it". (Sheikh Kassem Bader, Druze, Nabi Sabaln, 9.6.00).

D. Who or what is the punishing agent in response to not respecting the sacred tree?

1. God (**24**, only 4 Druze)

2. The Welli/prophet himself to whom the tree is dedicated (**32**, 17 of whom are Druze).

3. The axe that was used to cut down the tree (**7**, all Druze)

4. Snakes are the protectors of sacred trees (**8 **Druze, **2 **Muslims).

### Miracles that occurred under or near a sacred tree

1. The dead body of an impure girl that/who was buried under the/a sacred tree was cast out of its grave by the miracles of Welli to whom the tree is dedicated. (The sacred oak of Sheikh Abu Arus in the Druze village of Jat; a common story in the village. (**6**)).

2. Sacred trees swallowed innocent people/prophets to protect them from their enemies. (**6**, all Druze)

3. "One day a blind man sat under the sacred tree. There was a strong wind and a twig snapped off and struck the person and wounded him. The man wiped away the blood and then he got back his sight". (Haj Khalfalla Khalil, Muslim, Sheikh Danun, 24.8.04).

4. "The locusts did not touch the sacred tree although they devoured all other plants. The tree has a scent like jasmine, and that deterred the locusts". (Tawafiq Amashe, Druze, Mas'ade, 12 Dec. 2001)

5. Every Friday, stones roll under the sacred tree of U'm Ayash, in the village of Ibtin (Western Galilee). (A well known story in the village; (**6)**, although today there are no stones underneath the tree).

6. A man lost all his chickens which escaped from their cage He prayed under the tree and lit a candle; all the fowls then returned by themselves to the cage (Hady Samiyah, Muslim, Mazra'a, 24.8.04).

7. The corpse of an important man was temporarily buried under a sacred tree. It did not decay when it was removed to a permanent grave. A woman who saw the dead body went blind on the spot. (Sheikh Nur Rifaee, 16 June 2000; Majdal Kurum, Lower Galilee, the tale concerns his grandfather and it happened in 1936).

8. People who dared to steal property under the sacred tree walked or rode the whole night long, but the following morning they found themselves under the very same tree. (**12**).

### Breakdown of machinery

When a road was being constructed near the sacred tree of Sheikh Saris (5 km west of Majdal Kurum) the bulldozers got stuck. The villagers called in a religious leader who "contacted" the Welli. This spirit instructed him "to divert the road 70 paces from him and than everything will be settled peacefully". This was done, and there were no more problems (**9**).

The traditions on breakdown of mechanical tools which were used to remove sacred trees are so robust that even when large trees were intentionally preserved in the construction of new roads people still tell stories about such stoppages/breakdowns On the way to Tiv'on, a town near Haifa, there is a huge *Pistacia atlantica *in the middle of a highway. No less than 21 informants told us that the saint who was buried under the tree made the road builder divert the original route because of accidents or damage to the bulldozers working near the tree. The plain truth is that Salman Abu Rucan, then (in 1985) an inspector of Nature Reserve Authorities (NRA), asked the Public Works Department to preserve the tree (Salman Abu Rucan (12 March 1999, personal communication). A similar story is told about the sacred Oak (*Quercus calliprinos*) near the grave of Sheikh Ajami on the highway to Jerusalem (Bab el Wad). When the tractors approached the place they got mired down. The vehicles were repaired, but they got stuck again. Work on the road continued only after displacement of the original route several metres around the grave and the tree (**7**). In this case an NRA inspector prevents/prevented the uprooting of the tree and the place from being ruined. (The late Yigal Ronen, personal. Communication, 10 March 1970).

## Discussion

### The supernatural characters of the sacred trees

#### The tree as the abode of the soul of a saint

The most common "function" of the sacred trees in the Middle East is to serve as the abode of the spirit/soul of a saint (Welli, [42]; passim). Curtiss [[142]:75,77,79], noted, regarding the status of the saints in the Muslim world, " ... orthodox Moslems insist that the saints are only mediators that a worshipper asks his Welli to intercede for him with God... These saints are really departed spirits, connected with some particular shrine, chosen because they revealed themselves there in the past, and where they were wont to reveal themselves now to tmose who seek for favour... The worship of saints is like that of the ancient Baalim. They are the deities whom people fear, love, serve and adore". Canaan [[40]:151] adds, along the same lines "The present-day peasant does not venerate the trees themselves but the divine power which acts in them and which is derived from a godly person whose soul is supposed to inhabit the shrine, tomb, cave or spring with which they became associated. Often these holy men have appeared in the tree itself or near by ". This attitude explains the source of the supernatural nature attributed to the sacred trees (see below).

#### Unusual lights/voices

Hanauer [[58]:216] reports, "On Thursday evenings especially, one sometimes sees these trees lit up, and can hear snatches of sacred instrumental music proceeding from there. It is a sign that the saints are observing a festival and exchanging visits". Lights and voices around sacred trees were already reported from Palestine as well as in other countries (Table 1). Lights were seen also on sacred graves (Palestine, 16^th ^century, [59]:179).

In Teutonic traditions, old churches in sacred groves originated/were built where miraculous lights had been observed, arranged in the shape of the future church. Many of the old churches are said to owe their origin to such lights being seen in a grove or wood during the night. [[60]: IV: 1313].

#### The tree does not burn?

Sacred trees, do not burn: this belief goes back to the theophany of God to Moses (*Exodus 3:2 "And the angel of the Lord appeared unto him in a flame of fire out of the midst of a bush: and he looked*, ***and, behold, the bush burned with fire and the bush was not consumed."***

Robertson-Smith [[61]: 193–194] mentioned several historical cases in which lights and fire were seen around sacred trees which were the focus of ancient Semitic religious ceremonies.

This old tradition may serve as indirect evidence/an indication of the deeply rooted belief that sacred trees do not burn; it is still very common, in Israel, even today.

#### Miracles related to the tree

The motif of returning stolen property placed near a sacred grave and or a tree is already known. Rabbi Moshe of Bassula (14^th ^century) describes the grave of a righteous person "...and it is a grave of marble and a large stone cover (above it). People maintain, certainly, that about forty years ago the Ishmaelites took this cover to make a grinding stone. Many people carried this stone to a distant place and in the following morning found the stone (back) on its grave" [[62]:49]. The same traveller reported that a man loaded onto his donkey some wood which had fallen beneath a sacred tree; the animal went round and round the tree and was unable to leave the place till the wood was unloaded. [[63]:154].

The motif of the sacred tree as protector of innocent people appears in a Druze legend from our region [[64]:50]. There are stories about saints, leaders, and kings [England, [65]:115], saints [Ireland, [20]:39], and nuns [Ireland, [20]:35; [66]:70] who were under immediate threat of death, and then the tree provided them with shelter and even swallowed them; their lives were saved [see also [67: 124]].

The story of the casting of a body out of its grave is also told by the Bedouin in the Negev. Bar Tsvi et al. [[68]:84–85] tell about an unworthy person who was buried near the grave of a righteous man. His body was flung out of its grave several times until at last he was interred outside the cemetery (see below on the similar powers of graves and sacred trees).

#### Bleeding trees

The belief that blood flows from trees has ancient roots. Ovid [[39]:738–878] tells of Erysichton, king of Thrace, who commanded that a sacred oak dedicated to Demeter be cut down. The appeal of the Dryad that lived in the tree was in vain. The tree was chopped down, and she was doomed to die with the ruin of her abode. The revenge of Demeter, the goddess of fruit, crops and vegetation, was immediate and singularly cruel. The king was condemned to an eternal and insatiable hunger. Stories of bleeding trees are quite common (Table 1). In Israel *Ziziphus spina christi *is especially respected because of its red sap, which looks like blood; it appears when the tree is hurt [[68]:89–90; [69]:6].

#### Unusual dimensions/fruit/leaves

Jewish travellers who visited holy places in Palestine, especially graves of righteous Jewish historical figures, left "evidence" of miraculous sacred trees, attributing unusual characters to the plants. An anonymous traveller from Candia (Crete) wrote in 1481 about the grave of Hanina ben Dossa: "...on the grave there are two trees whose shadow is seen from a distance such a mile" [[59]:88]. Shim'on Berman visited the oak of Mamre in 1870 and stated that "...the tree is so intricate that a thousand people can stand underneath" [[70]:82). Another traveller (Rabbi Shmuel Ben Shimson) of the 13^th ^century described a holy grave "on which there is a big *Pistacia *which has the shape of a lion" [[45]:119]. Rabbi Moshe of Bassula (16^th ^century) described the tree over the grave of Yonathan Ben Uziel in Amuqa (there is today a sacred tree on the spot but it is not certain that it is the same one): "There is nothing like this tree in its thickness and the width of its branches and beauty" [[63]:141]).

Old sources from Ireland [20] on sacred trees describe one that has silver and gold leaves [20:19], bears three kinds of fruit (acorns, apples, and hazel nuts, [20:18, 41]), it all being of extraordinary size [20:17], with large, unusual, leaves [20:19], and having the ability to recover immediately after any injury [20:37]. Our informants (**4**) report on the unusual sweetness of the acorns of the oak, (Quercus *calliprinos*, the most common sacred tree) and on the fruit of *Ziziphus spina christi *which are not infected by caterpillars in comparison to "non-sacred trees" of the same species.

#### Vital powers/trees as oracles

Throughout history, and in many cultures, sacred trees were regarded as omens and oracles, as well as soothsayers that may speak in human voices [[67]:245, 247; [6]:24; [19]: passim; [61]:195; [71] II: 17–18; [13]: passim; [50]: passim; [27]:275–278]. This issue was mentioned in the classical mythology of Greece [[72]:passim; [17]: passim; [50]:58; [27]:274], Rome [50]:59; [27]:274], as well as the Bible [Judges,9:8–15, Jotham's parable,; for further discussion see [61]:195; [67]:246–247; [73]: passim]. Doughty [[74]: II: 209] mentions an Arabic legend regarding trees through which a soothsayer, a spirit, speaks. Nowhere did we find this animistic element of tree worship [[50]:152; [67]:245] in Israel. It seems that the present-day reports are all related to polytheistic religions.

One may thus conclude that the local repertoire of miracles and other supernatural characters attributed to sacred trees in our region (see also Tables 1 and 2) are, more or less similar to those in Europe as well as in our region.

### The supernatural powers of sacred trees

#### Punishment of whoever violates the tree

All over the world sacred trees are protected by a system of taboos and ceremonies which were developed to prevent any damage [[13]: passim; [14]: passim; [50]:passim; [75]: 35; [76]: 350;351; [77]::1578; [78]:311; [79]:5, 18; [80]:224; [55]:49; [60]:712; [81]:9; [82]:699; [83]:6;, Millar et al 1991, 75:35;). These trees are regarded as the abode of supernatural beings/gods/souls/demons [[84]:52; [28]:316]] and any harm to such abodes are to be heavily punished. Cutting down sacred trees is regarded as a particularly serious offence against the supernatural element because such an act leaves the spirit homeless [[33]: I, 133; [85]:11 see also [42]]. There is thus a need to repatriate these supernatural beings by means of special ceremonies [[33]:133–137).

The punishing agent in response to a sacrilege against a sacred grave [[86]:106–107] or a tree maybe the saint himself to whom the tree is dedicated [Palestine: [43]:215; [40]:36; [87]:66; India: [88]:90 ], the deity resident in the tree [West Africa: [29]:243], the wood spirit [Turkey: [15]:215], or the fallen tree itself [India: [55]:278; Japan: [25]:37; Russia: [89]:188). The mechanical saw that was used to cut down the tree killed the offender (Rajasthan: [90]:219). According to Jaussen [91:333], "No Arab [in Transjordan] would dare to cut off a bough ... he should be immediately struck by the spirit of the saint (Welli) that resides in the tree and has made it his domain."

Lake [[92]:13] noted that in many cultures tree and serpent worship are closely related, and these things are deified as symbols of the reproductive powers of nature and are often visible representations of God. The connection between the tree of life, sacred/holy trees, and snakes is deep-rooted in many cultures, such as Old Sumer [[93]:279; [94]:179], Babylon [[95:118]; [96]:128). The Bible (Genesis 3, 1–4), Old Syria [[97]:442), Greek mythology [[98]:110; [99]:52–54I], as well as present-day India [[100]:241; [101]: passim; [102]:279; [85]:61]. The snake as a divine agent of punishment is well known from the Bible (Numbers 21, 6); Job, 20:16; Isaiah, 14:29; Jeremiah, 8:17; and Amos, 5:19). In Ancient Greece, snakes were frequently depicted round sacred trees [[103]:18; [104]:16) or as inhabitants of sacred groves [[50]:62, also in Rome [50]: 70–71].

Snakes were known as legendary protectors of treasures [[100]:262,270], homes [[100]:269,271; [96]:116 note no.7; [15]:224], and temples [100]:262,271]. It is not surprising to find snakes regarded as the protectors of sacred trees, for example, in Greek mythology [[98]:11; [17]:33; [105]:250], Sumer (Piper:,1989,19,:82); India [[102]:26; [106]:385; [88]:103, [107]:278], Zimbabwe [[108]:200], Mozambique [[109]:14]; Japan [[25]:35], Ecuador [[21]:66], and Sinai [110]:185] as mentioned by some of our informants (especially Druze).

In some cases the punishment for the violation of sacred tree is parallel to the deed itself: the burning of property is the penalty for taking wood for fire or heating [[Palestine: [87]:66; [111]:44]; Ireland: [[20]:23,38,40], destruction of offender's the house as a response to taking wood for building or other forms of construction [[Palestine:[87]:66; Syria: [112]:16].

The belief that the axe that cut/fells the tree is the punishing agent appeared in Greek mythology. Halirrhothius, the son of Poseidon, was angered at Athena's victory over his father in the contest to be the tutelary divinity of Athens He sought to cut down the sacred olive, (at the acropolis) but the axe head struck him and he died [[113]: 1006; [114], 1.18]. Similar stories have been recorded from several countries such as India [90]:219], West Africa [29]:205], Ireland [12^th ^century, [20]:28) and Lithuania [15^th ^century, [115]: 37]).

### Granting a divine favour/cure/blessing

Objects near sacred graves "absorb the sanctity of the place and/or have magic powers in the Muslim world" [[116]:106] as well as in Christianity [[117]:1086] and are used later by the believers. It is not surprising to find "seepage" of the supernatural powers of sacred graves to sacred trees, so some functions of the saint's grave such as: granting *barakeh*, property protection (Table 2), swearing, and taking vows were transferred to the sacred tree (even in the absence of the grave) or granted by the saint himself associated with the tree (see below).

The manner in which clothes are tied to a sacred tree to transfer personal troubles [[118]: passim; [41]: passim, and references therein) or using objects that "absorb" the supernatural powers of the tree are typical examples of contact magic. Frazer [[119]:18] explained "things which have once been in contact with each other at a distance after the physical contact has been severed. Practically all these manners are methods of "conveying the divine effluence" [[118]:467] from the tree to the devoted people. In this way the supernatural being, which is connected with the sacred tree, has the power to grant petitions (e.g. "wishing trees") which is common worldwide (Table 2).

#### Breakdown of machinery

Stories about the breakdown of bulldozers working too close to a grave containing the remains of saints are known from the Galilee in the North of Israel [[111]:50; [44]:284, and 23 of our informants] and from the Sinai in the South of the country [[110]:65], as well as from India [[90]:220]). This is another modern example of the reprisal powers of the sacred man against the sacrilege of/intrusion into his personal territory.

#### Sacred trees as sources of medical cures?

Sacred trees are believed to have magic curative powers in pagan Europe [[14]:42–45; [35]:277; [133], II: 169–193]. In Israel, even species of some plants or parts of it that are not known as having medicinal properties, such as the leaves of a sacred oak (*Quercus calliprinos*), are regarded as omnipotent forms of medication when administrated externally as a poultice (**6**) or as a decoction (**8**). Leaves of a "secular" oak are not used for healing. Clearly, the leaves acquire the healing powers when granted by saints; just as actual medicinal plants gathered in the vicinity of the sacred tree are more potent than their conspecifics (**5**). Most of the uses of sacred trees for divine blessings or cures or as talismans (Table 2) seem based on magical contact. The extensive use of rags placed on sacred trees as agents of illness being transferred to the tree is noteworthy [[41] and references therein].

### Circumambulation around tree/graves

One way which claims to cure sick animals via a sacred tree is carried out by circumambulations of the animals around it several times. Circumambulations around sacred objects are well known in the Muslim world. Such objects are the Ka'ba in Mecca [[28]:287; [116]:267; [120]:111] as well as saints' shrines or graves [[40]:114; [116]:267; [120]:111). The same applies to the Christian realm [[116]:267]. Sometimes the references mention specifically sacred trees, such as in India [[30]:73; [88]:40] or in Poland [17^th ^century, [121]:403].

The purpose of the ancient and widespread rite of circumambulating a sacred object, tomb, or building is for those encircling the object to acquire some of the *barake *or healing powers of the shrine or the object circumambulated [[40]:114; [120]:111). Ritual dances around sacred trees in Lebanon to ensure good harvests are mentioned by Sessions [[122]:9].

Hasluck [[116]:267) explains the circumambulating as "... designed to secure the maximum amount of blessing from the sacred object in question by allowing all sides of it to act on the worshipper. It is thus a diluted form of contact with relics", which is so common in the Christian world [[8]: passim].

The practice of circumambulating trees still exists today, as found in the present study, but only to cure ill domestic animals. Sometimes the circumambulating could take place around a sacred place (**6 **informants). This form exists among the Bedouin in the Negev [[68]:91]. In our survey this practice was found almost exclusively in the Druze sector.

### Deposition of valuables on a sacred grave

Saints' graves throughout the Muslim world enjoy godly/divine protection, such graves were thus used as a place to deposit valuables, animals, agricultural machinery and even domestic animals and harvests [[40]:102–103; [28]291–292; [123]:7; [124]:95; [125]:1879; [126]:27; [127]:219]. People feared the revenge of the saint [[126]:27], who was regarded as the keeper of the sacred place himself [[128]:179; [43]:215–216; [14:] 42; [40]:102; [[68]:52]. Depositing properties within the domain of the saint (Mosque, Makkam (=holy local shrine), grave) is known also from Morocco [[129]:240]. Marcus tells about the strong belief in the efficacy of a certain saint: "One important theme of these stories concerns the mysterious return of stolen objects ....with the saint calling upon God to punish those who act wrongfully".

The Bedouin and peasants used to leave tents and some of their equipment under a sacred tree during their wanderings in the belief that the saint would safeguard them [[130]:35; [45]:154; [131]:53]. The punishments of the offenders against the sanctity of sacred graves and of sacred trees are similar (16 informants).

It seems that this manner has now disappeared in northern Israel although it is still flourishing in the oral tradition.

### Punishable deeds associated with sacred trees

According to Table 3, one can see, more or less, the same punishable deeds in relation to sacred trees/groves/forest worldwide. Only two items, disturbing praying people (only Druze) and theft of objects deposited under are tree are, as far as the author is aware, limited to our region the Middle East.

The list of the various limitations and prohibitions in so many diverse cultures may reflect the deep rooted veneration of trees throughout the course of history as well as in our own day.

### Punishments following the violation of sacred trees

The list of punishments following the violation of sacred trees/groves/forests (Table 4) shows that all the data recorded by us from Israel are already known from our region and/or worldwide. The magnitude of belief in the supernatural powers of the sacred trees is reflected by the high frequency of the data concerning serious punishments (Table 4). There are some noticeable quantitative variations among the three groups studied, especially concerning the death punishment, divine punishment and heart of a family member. We cannot see any trend in these differences and we cannot offer any explanation for it. The data shown in Table 4 serve also as clear evidence for the ubiquitous distribution of tree worship.

### Use of wood for religious purposes

Wood from sacred trees may be used, without fear of punishment, for religious purposes such as at festivals to honour the saint [Palestine: [40]:36; [126]:28; Egypt: [132]:17; Turkey: [116]:250; [133]:327); for sacrifices to the gods [Ancient Greece: [36]:442; [54]:174; [37]:336; [134]:154; [37]:337], for rites of passage [Sierra Leone: [78]:311], as offering to deities [India: [135]:330], for religious purposes [Indonesia: [136]:45; India [137]:345; India: [138:105]; [139]:67] or for the mosque or coffin making [Turkey: [15]:213]. Wood from a sacred forest, especially for religious purposes, may be used after a special prayer [46]:56; 9 of our informants] or after requesting permission from the deities, as in ancient Rome [140]:139–140; [36]:442] and India [100]:240; [55]:277; [141]:66, 68].

### Sacred trees and graves

In the Middle East, as in North Africa, a saint's grave is closely associated with a sacred tree; trees beneath which saints are buried are regarded as "sacred trees" [[142]:93; Cannan: [40]: passim; [42] passim]. The identification of the sacred tree with the saint's grave imparts to it, explains the miraculous and magical powers of the holy man [[67]:264; [142]:94; [40]:255–262; [116]:176–177]. Thus it is not surprising that the tree has the same powers as the grave, both of which are the abode of the souls of the Welli.

According to Wilson [[126]:27], people "fully believed that should they swear by one of these (the revered) shrines to do, or not to do, any certain thing, and should they be false to their oath, some fearful calamity would overtake them". Suspected people would swear on the grave of the Welli as a test of their purity (, 8 Muslim informants). People would swear under a sacred tree (19 Muslim informants) as well as in the Makam (the shrine of a Welli) or on his grave [[143]:442; [40]:127–130, 11 Muslim informants].

Objects near sacred graves "absorb" the sanctity of the place and/or havie magic powers in the Muslim world [[116]:106] as well as in Christianity [[117]:108] and are used later by the believers. Thus it is not surprising to find "seepage" of the supernatural powers of the sacred graves to the sacred trees.

In the Muslim world the worshipping of saints is very popular [[129]:456–457; [120]: 103–108]. According to Goldziher [[28]:290–201], "The primary function of the veneration of saints in Islam is to satisfy the instinct to look up to perfections within the human sphere which are worthy of veneration and admiration, the possessors of which are not only exercising the highest virtue and sanctity but have also the power – on behalf of those who trust in them – which appear impossible, things we call 'miraculous' ". Wilson [[126]:27], even noted (regarding the peasants of Palestine), "The Moslems stand in great awe of these saints, especially of the more famous of them, and often really fear them more than they fear God". People sought the healing power and the blessings that the saints possess [[120]:104; [129]:457]. They are regarded as mediators between God and the people [[129]:459]. Saints have the divine powers of granting *barake*, a kind of blessing that Westermarck [[26:79]) defined as "a wonder-working force of predominantly beneficial character".

### Forgiveness and atonement

In the Middle East one who offends against a sacred tree may ask for personal forgiveness from the Welli to whom the tree is dedicated (29 informants). The first stage is to return the stolen parts (of the tree or other goods) to the tree [also in India: [90]:221; [139]:67] and to sacrifice a sheep and to give the meat to the needy; the person will then be forgiven by the Welli and the punishment averted. This pattern of punishment for the violation of sacred trees, begging forgiveness, atonement, and release from the penalty is already known from Palestine [[43]:215], the Sinai [110]:184], and India [[90]:219]. Sometimes there is a requirement to deliver an offering or pay a ransom (see Table 4). In Israel we recorded no case of ransom or payment; this aspect seems related only to communities with special laws protecting the sacred groves and the gods that live in them.

In some cases special prayers, ceremonies, fines, offerings, or sacrifices are practised to ask permission or forgiveness of the wood gods before a tree can be cut down or materials are extracted from the sacred area, for example, Rome [[50]:67; [144]:124; [32]:99); Egypt: [46:56]; Bangladesh: [145]:177; Central Africa: [47]:229–230; [146]:317; East Africa: [47]:229; [29]:205; Indonesia [49]:313; [136]:26; Japan [25]:37; India: [106]:384]. These actions might take place after the operation, for example, in India [90]:22]; Central Africa [47]:229; Japan [25]:36, See also [14]: passim]. This is done to prevent the expected malignant response of the deities, saints, or gods. Such "preventive" actions were never recorded in Israel.

### Monotheistic vs. polytheistic sacred trees

The relationship between people, tree worship, and punishment by the sacred tree for misdeeds evinces a kind of a repeated pattern. In the Middle East, as well as in Europe, tree worship today is practised by individuals making personal petitions. Tree worship is, by no means, a part of the official monotheistic governing religion [[42]. Punishment falls upon the offender himself, his family or his property (Table 4). But, in many polytheistic religions, tree worship is established on a community basis which is influenced by the divine entity to which the trees are dedicated (see below).

A possible reason for this putative pattern seems to be related to the meaning of the trees in the life of the community in their present habitat. Most monotheistic tree worship is confined to temperate zones; and the "unit of veneration" nowadays is mainly a single tree. The tree/wood/grove has almost no importance in the religious/economic/social life of the community.

In many polytheistic religions, most of which still prevail in areas of tropical or semitropical vegetation, the "unit of veneration" is the sacred wood/grove/forest, which is the centre of well-established religious ceremonies; and its veneration is a part of the regular worship, and led sometimes by official figures. The wood/forest/grove is essential to their very existence (e.g., source of medicinal plants, food, fuel and/or protection of watersheds), or was so until recently. The systems of punishment were established as a means to protect the wood/forest/grove resources from over-exploitation as an essential resource of the community. Any wilful harm to the trees was regarded as a direct act of sacrilege against the supernatural power that is the benefactor of the community and which may punish the whole community in revenge. Punishment occurs frequently against the whole community, and includes calamities such as fire, flood, or plague. Thus, there is a need to repatriate the supernatural power who guards the community and to whom the trees are dedicated. This pattern has been recorded fully or partly in: Vietnam: [147]:113; Kenya: [148]:89; Mozambique:[109]:14; Laos: [149]:324; China: [150]:352; [151]:6; [152]:131–132; India: [153]:8; [137]:345; [141]:66–68; [154]:386; [153]:8; [155]:315–319; [106]:384; [156]:96; East Africa: [48]:414,432; Côte d'Ivoire: [157]:370; Nigeria: [158]:290,292,293; Ghana [159]:366; [160]:159; [161]:90–99; Vietnam: [162]:113.

## Conclusion

In the discussion of the reason for the sanctification of trees [[40]:30, 38; [42]] the close relation with graves of Muslim saints (Wellis) has been shown. The spirit of the Welli dwells in his grave or in a tree dedicated to him. It is not surprising to see the close similarity between the miraculous powers of the holy grave and the sacred trees. In the Muslim sector the close similarity between the ability to punish and the protective properties of graves and sacred trees is clearly evident. In both cases the protective power is the Welli's spirit, which the people admire and fear.

The Druze believes in the transmigration of souls: a person's body is a kind of clothing for the soul and, with death, the soul passes to the body of a newborn child [[57]:60]. The Druze never considers sacred trees as an abode for the souls of righteous figures of righteous figures' souls, and certainly do not relate trees to graves [42]. It was, therefore, unexpected to see that even the Druze ascribe supernatural powers to sacred trees. Their fear and admiration of such trees are of the same magnitude as in the Muslim sectors [(Table 3, [41]; [42]]. While the Muslims credit the miraculous powers (e.g., the trees' immunity to fire) to the souls of Wellis or of God, the Druze ascribe them to their prophets or religious leaders themselves.

A comparison with the Christian world shows a clear similarity between the miracles performed by the sacred trees (via the spirit of the Welli) and the miraculous powers of saints and their trees. We may recall that many pagan sacred trees were Christianized and dedicated to saints [[32]:107–108; [20]:34; [50]:162; [60]: I, 86–87] while in the Muslim world the old traditions of sacred trees were not eradicated: the tree spirits were replaced by the souls of Wellis. [Palestine: [40]:151; Morocco: [26]:97]. Not surprisingly, the old pagan traditions of miraculous powers of sacred trees filtered into the Christian as well as the Muslim world. The Druze adopted most of the same traditions but on a different religious basis.
